# A Brief Review of Generalized Entropies

**DOI:** 10.3390/e20110813

**Published:** 2018-10-23

**Authors:** José M. Amigó, Sámuel G. Balogh, Sergio Hernández

**Affiliations:** 1 Centro de Investigación Operativa, Universidad Miguel Hernández, Avda. de la Universidad s/n, 03202 Elche, Spain; 2 Department of Biological Physics, Eötvös University, H-1117 Budapest, Hungary; 3 HCSoft Programación S.L., 30007 Murcia, Spain

**Keywords:** generalized entropy, Tsallis, Rényi, Hanel–Thurner exponents, non-stationary regime

## Abstract

Entropy appears in many contexts (thermodynamics, statistical mechanics, information theory, measure-preserving dynamical systems, topological dynamics, etc.) as a measure of different properties (energy that cannot produce work, disorder, uncertainty, randomness, complexity, etc.). In this review, we focus on the so-called generalized entropies, which from a mathematical point of view are nonnegative functions defined on probability distributions that satisfy the first three Shannon–Khinchin axioms: continuity, maximality and expansibility. While these three axioms are expected to be satisfied by all macroscopic physical systems, the fourth axiom (separability or strong additivity) is in general violated by non-ergodic systems with long range forces, this having been the main reason for exploring weaker axiomatic settings. Currently, non-additive generalized entropies are being used also to study new phenomena in complex dynamics (multifractality), quantum systems (entanglement), soft sciences, and more. Besides going through the axiomatic framework, we review the characterization of generalized entropies via two scaling exponents introduced by Hanel and Thurner. In turn, the first of these exponents is related to the diffusion scaling exponent of diffusion processes, as we also discuss. Applications are addressed as the description of the main generalized entropies advances.

## 1. Introduction

The concept of entropy was introduced by Clausius [1] in thermodynamics to measure the amount of energy in a system that cannot produce work, and given an atomic interpretation in the foundational works of statistical mechanics and gas dynamics by Boltzmann [2,3], Gibbs [4], and others. Since then, entropy has played a central role in many-particle physics, notoriously in the description of non-equilibrium processes through the second principle of thermodynamics and the principle of maximum entropy production [5,6]. Moreover, Shannon made of entropy the cornerstone on which he built his theory of information and communication [7]. Entropy and the associated entropic forces are also the main character in recent innovative approaches to artificial intelligence and collective behavior [8,9]. Our formalism is information-theoretic (i.e., entropic forms are functions of probability distributions) owing to the mathematical properties that we discuss along the way, but can be translated to a physical context through the concept of microstate.

The prototype of entropy that we are going to consider below is the Boltzmann–Gibbs–Shannon (BGS) entropy,

(1) $$S_{BGS}\left(p_{1},\ldots ,p_{W}\right)=k\sum_{i=1}^{W}p_{i}\ln\frac{1}{p_{i}}=-k\sum_{i=1}^{W}p_{i}\ln p_{i}.$$

In its physical interpretation, $k=1.3807\times 10^{-23}$ J/K is the Boltzmann constant, *W* is the number of microstates consistent with the macroscopic constraints of a given thermodynamical system, and $p_{i}$ is the probability (i.e., the asymptotic fraction of time) that the system is in the microstate *i*. In information theory, *k* is set equal to 1 for mathematical convenience, as we do hereafter, and $S_{BGS}$ measures the average information conveyed by the outcomes of a random variable with probability distribution $\left\{p_{1},\ldots ,p_{W}\right\}$. We use natural logarithms unless otherwise stated, although logarithms to base 2 is the natural choice in binary communications (the difference being the units, nats or bits, respectively). Remarkably enough, Shannon proved in Appendix B of his seminal paper [7] that Equation (1) follows necessarily from three properties or axioms (actually, four are needed; more on this below).

BGS entropy was later on generalized by other “entropy-like” quantities in dynamical systems (Kolmogorov–Sinai entropy [10], etc.), information theory (Rényi entropy [11], etc.), and statistical physics (Tsallis entropy [12], etc.), to mention the most familiar ones (see, e.g., [13] for an account of some entropy-like quantities and their applications, especially in time series analysis). Similar to with $S_{BGS}$, the essence of these new entropic forms was distilled into a small number of properties that allow sorting them out in a more systematic way [13,14]. Currently, the uniqueness of $S_{BGS}$ is derived from the four *Khinchin–Shannon axioms* (Section 2). However, the fourth axiom, called the *separability* or *strong additivity axiom* (which implies additivity, i.e., $S\left(A_{1}+A_{2}\right)=S\left(A_{1}\right)+S\left(A_{2}\right)$, where $A_{1}+A_{2}$ stands for a system composed of any two probabilistically independent subsystems $A_{1}$ and $A_{2}$), is violated by physical systems with long-range interactions [15,16]. This poses the question of what mathematical properties have the “generalized entropies” satisfying only the other three axioms. These are the primary candidates for *extensive* entropic forms, i.e., functions *S* such that $S\left(B_{1}\cup B_{2}\right)=S\left(B_{1}\right)+S\left(B_{2}\right)$, the shorthand $B_{1}\cup B_{2}$ standing for the physical system composed of the subsystems $B_{1}$ and $B_{2}$. Note that $B_{1}\cup B_{2}\neq B_{1}+B_{2}$ in non-ergodic interacting systems just because the number of states in $B_{1}\cup B_{2}$ is different from the number of states in $B_{1}+B_{2}$. A related though different question is how to weaken the separability axiom to identify the extensive generalized entropies; we come back briefly to this point in Section 2 when speaking of the composability property.

Along with $S_{BGS}$, typical examples of generalized entropies are the *Tsallis entropy* [12],

(2) $$T_{q}\left(p_{1},\ldots ,p_{W}\right)=\frac{1}{1-q}\left(\sum_{i=1}^{W}p_{i}^{q}-1\right)$$

(q∈R, q≠1, with the proviso that for q<0 terms with $p_{i}=0$ are omitted), and the *Rényi entropy* [11],

(3) $$R_{q}\left(p_{1},\ldots ,p_{W}\right)=\frac{1}{1-q}\ln\left(\sum_{i=1}^{W}p_{i}^{q}\right)$$

(q≥0, q≠1). The Tsallis and Rényi entropies are related to the BGS entropy through the limits

$$\lim_{q\to 1}T_{q}\left(p_{1},\ldots ,p_{W}\right)=\lim_{q\to 1}R_{q}\left(p_{1},\ldots ,p_{W}\right)=S_{BGS}\left(p_{1},\ldots ,p_{W}\right),$$

this being one of the reasons they are considered generalizations of the BGS entropy. Both $T_{q}$ and $R_{q}$ have found interesting applications [15,17]; in particular, the parametric weighting of the probabilities in their definitions endows data analysis with additional flexibility. Other generalized entropies that we consider in this paper are related to ongoing work on graphs [18]. Further instances of generalized entropies are also referred to below.

Let us remark at this point that $S_{BGS}$, $T_{q}$, $R_{q}$ and other generalized entropies considered in this review can be viewed as special cases of the (h,ϕ)-entropies introduced in [19] for the study of asymptotic probability distributions. In turn, (h,ϕ)-entropies were generalized to quantum information theory in [20]. Quantum (h,ϕ)-entropies, which include von Neumann’s entropy [21] as well as the quantum versions of Tsallis’ and Rényi’s entropies, have been applied, for example, to the detection of quantum entanglement (see [20] and references therein). In this review, we do not consider quantum entropies, which would require advanced mathematical concepts, but only entropies defined on classical, discrete and finite probability distributions. If necessary, the transition to continuous distributions is done by formally replacing probability mass functions by densities and sums by integrals. For other approaches to the concept of entropy in more general settings, see [22,23,24,25].

Generalized entropies can be characterized by two scaling exponents in the limit W→∞, which we call Hanel–Thurner exponents [16]. For the simplest generalized entropies, which include $T_{q}$ but not $R_{q}$ (see Section 2), these exponents allow establishing a relationship between the abstract concept of generalized entropy and the physical properties of the system they describe through their asymptotic scaling behavior in the thermodynamic limit. That is, the two exponents label equivalence classes of systems which are universal in that the corresponding entropies have the same thermodynamic limit. In this regard, it is interesting to mention that, for any pair of Hanel–Thurner exponents (at least within certain ranges), there is a generalized entropy with those exponents, i.e., systems with the sought asymptotic behavior. Furthermore, the first Hanel–Thurner exponent allows also establishing a second relation with physical properties, namely, with the diffusion scaling exponents of diffusion processes, under some additional assumptions.

The rest of this review is organized as follows. The concept of generalized entropy along with some formal preliminaries and its basic properties are discussed in Section 1. As way of illustration, we discuss in Section 3 the Tsallis and Renyi entropies, as well as more recent entropic forms. The choice of the former ones is justified by their uniqueness properties under quite natural axiomatic formulations. The Hanel–Thurner exponents are introduced in Section 4, where their computation is also exemplified. Their aforementioned relation to diffusion scaling exponents is explained in Section 5. The main messages are recapped in Section 6. There is no section devoted to the applications but, rather, these are progressively addressed as the different generalized entropies are presented. The main text has been supplemented with three appendices at the end of the paper.

## 2. Generalized Entropies

Let P be the set of probability mass distributions $\left\{p_{1},\ldots ,p_{W}\right\}$ for all W≥2. For any function $H:P\to R^{+}$ ($R^{+}$ being the nonnegative real numbers), the *Shannon–Khinchin axioms* for an entropic form *H* are the following.

**SK1**  *Continuity*. $H(p_{1},\ldots ,p_{W})$ depends continuously on all variables for each *W*.
**SK2**  *Maximality*. For all *W*,
  $$H\left(p_{1},\ldots ,p_{W}\right)\leq H\left(\frac{1}{W},\ldots ,\frac{1}{W}\right).$$
**SK3**  *Expansibility*: For all *W* and 1≤i≤W,
  $$H\left(0,p_{1},\ldots ,p_{W}\right)=H\left(p_{1},\ldots ,p_{i},0,p_{i+1},\ldots ,p_{W}\right)=H\left(p_{1},\ldots ,p_{i},p_{i+1},\ldots ,p_{W}\right).$$
**SK4**  *Separability* (or *strong additivity*): For all W,U,
  (4) $$\begin{array}{c} H(p_{11},\ldots ,p_{1U},p_{21},\ldots p_{2U},\ldots ,p_{W1},\ldots ,p_{WU})\;\\ =H\left(p_{1\cdot },p_{2\cdot },\ldots ,p_{W\cdot }\right)+\sum_{i=1}^{W}p_{i\cdot }H\left(\frac{p_{i1}}{p_{i\cdot }},\frac{p_{i2}}{p_{i\cdot }},\ldots ,\frac{p_{iU}}{p_{i\cdot }}\right), \end{array}$$
  where $p_{i\cdot }=\sum_{j=1}^{U}p_{ij}$.

Let $\left\{p_{11},\ldots ,p_{1U},p_{21},\ldots p_{2U},\ldots ,p_{W1},\ldots ,p_{WU}\right\}$ be the joint probability distribution of the random variables *X* and *Y*, with marginal distributions $\left\{p_{i\cdot }:1\leq i\leq W\right\}$ and $\left\{p_{\cdot j}=\sum_{i=1}^{W}p_{ij}:1\leq j\leq U\right\}$, respectively. Then, axiom SK4 can be written as

$$H\left(X,Y\right)=H\left(X\right)+H(Y\left|X\right),$$

where $H(Y\left|X\right)$ is the entropy of *Y* conditional on *X*. In particular, if *X* and *Y* are *independent* (i.e., $p_{ij}=p_{i\cdot }p_{\cdot j}$), then $H(Y\left|X\right)=H\left(Y\right)$ and

(5) H(X,Y)=H(X)+H(Y).

A function *H* such that Equation (5) holds (for independent random variables *X* and *Y*) is called *additive*. Physicists prefer writing X+Y for composed systems with microstate probabilities $p_{ij}=p_{i\cdot }p_{\cdot j}$; this condition holds approximately only for weakly interacting systems *X* and *Y*.

With regard to Equation (5), let us remind that, for two general random variables *X* and *Y*, the difference I(X;Y)=H(X)+H(Y)−H(X,Y)≥0 is the mutual information of *X* and *Y*. It holds I(X;Y)=0 if and only if *X* and *Y* are independent [26].

More generally, a function *H* such that

(6) $$\begin{array}{c} H(p_{1}q_{1},\ldots ,p_{1}q_{U},p_{2}q_{1},\ldots ,p_{2}q_{U},\ldots ,p_{W}q_{1},\ldots ,p_{W}q_{U})\;\\ =H\left(p_{1},\ldots ,p_{W}\right)+H\left(q_{1},\ldots ,q_{U}\right)+\left(1-\alpha \right)H\left(p_{1},\ldots ,p_{W}\right)H\left(q_{1},\ldots ,q_{U}\right), \end{array}$$

(α>0) is called α-*additive*. With the same notation as above, we can write this property as

(7) H(X,Y)=H(X)+H(Y)+(1−α)H(X)H(Y),

where, again, *X* and *Y* are independent random variables. In a statistical mechanical context, *X* and *Y* may stand also for two probabilistically independent (or weakly interacting) physical systems. If α=1, we recover additivity (Equation (5)).

In turn, additivity and α-additivity are special cases of *composability* [15,27]:

(8) H(X,Y)=Φ(H(X),H(Y)),

with the same caveats for *X* and *Y*. Here, Φ is a symmetric function of two variables. Composability was proposed in [15] to replace axiom SK4. Interestingly, it has been proved in [27] that, under some technical assumptions, the only composable generalized entropy of the form in Equation (10) is $T_{q}$, up to a multiplicative constant.

As mentioned in Section 1, a function $F:P\to R^{+}$ satisfying axioms SK1–SK4 is necessarily of the form $F\left(p_{1},\ldots ,p_{W}\right)=kS_{BGS}\left(p_{1},\ldots ,p_{W}\right)$ for every *W*, where *k* is a positive constant ([28], Theorem 1). The same conclusion can be derived using other equivalent axioms [14,29]. For instance, Shannon used continuity, the property that H(1/n,…,1/n) increases with *n*, and a property called *grouping* [29] or *decomposibility* [30], which he defined graphically in Figure 6 of [7]:

(9) $$\begin{array}{ccc} H(p_{1},\ldots ,p_{W}) & = & H(\left(p_{1}+\ldots +p_{r}\right),\left(p_{r+1}+\ldots +p_{W}\right))\\ & & +\left(p_{1}+\ldots +p_{r}\right)H\left(\frac{p_{1}}{\sum_{i=1}^{r}p_{i}},\ldots ,\frac{p_{r}}{\sum_{i=1}^{r}p_{i}}\right)\\ & & +\left(p_{r+1}+\ldots +p_{W}\right)H\left(\frac{p_{r+1}}{\sum_{i=r+1}^{W}p_{i}},\ldots ,\frac{p_{W}}{\sum_{i=r+1}^{W}p_{i}}\right) \end{array}$$

(1≤r≤W−1). This property allows reducing the computation of $H(p_{1},\ldots ,p_{W})$ to the computation of the entropy of dichotomic random variables. According to ([15], Section 2.1.2.7), Shannon missed in his uniqueness theorem to formulate the condition in Equation (5), *X* and *Y* being independent random variables.

Nonnegative functions defined on P that satisfy axioms SK1–SK3 are called *generalized entropies* [16]. In the simplest situation, a generalized entropy has the *sum property* [14], i.e., the algebraic form

(10) $$F_{g}\left(p_{1},\ldots ,p_{W}\right)=\sum_{i=1}^{W}g\left(p_{i}\right),$$

with $g:\left[0,1\right]\to R^{+}$.

The following propositions are immediate.

(**i**) *Symmetry*: $F_{g}\left(p_{1},\ldots ,p_{W}\right)$ is invariant under permutation of $p_{1},\ldots ,p_{W}$.
(**ii**) $F_{g}$ satisfies axiom SK1 if and only if *g* is continuous.
(**iii**) If $F_{g}$ satisfies axiom SK2, then
  $$\sum_{i=1}^{W}g\left(p_{i}\right)\leq Wg\left(\frac{1}{W}\right)$$
  for all W≥2 and $p_{1},\ldots ,p_{W}$ with $p_{1}+\ldots +p_{W}$=1.
(**iv**) If *g* is concave (i.e., ∩-convex), then $F_{g}$ satisfies axiom SK2.
(**v**) $F_{g}$ satisfies axiom SK3 if and only if g(0)=0.

Note that Proposition (iv) follows from the symmetry and concavity of $F_{g}$ (since the unique maximum of $F_{g}$ must occur at equal probabilities).

We conclude from Propositions (ii), (iv) and (v) that, for $F_{g}$ to be a generalized entropy, the following three condition suffice:

(**C1**) *g* is continuous.
(**C2**) *g* is concave.
(**C3**) g(0)=0.

As in [16], we say that a macroscopic statistical system is *admissible* if it is described by a generalized entropy $F_{g}$ of the form in Equation (10) such that *g* verifies Conditions (C1)–(C3). By extension, we say also that the generalized entropy $F_{g}$ is admissible. Admissible systems and generalized entropies are the central subject of this review. Clearly, $S_{BGS}$ is admissible because

(11) g(x)=−xlogx,

0≤x≤1. On the other hand, $T_{q}$ corresponds to

(12) $$g\left(x\right)=\frac{1}{1-q}\left(x^{q}-x\right).$$

For $T_{q}$ to be admissible, Condition (C1) requires q≥0 and Condition (C3) requires q>0.

An example of a function $F:P\to R^{+}$ with the sum property that does not qualify for admissible generalized entropy is

(13) $$F\left(p_{1},\ldots ,p_{W}\right)=\sum_{i=1}^{W}{\left(p_{i}-\frac{1}{W}\right)}^{2}=\sum_{i=1}^{W}p_{i}^{2}-\frac{1}{W}.$$

Indeed, $g\left(x\right)={\left(x-\frac{1}{W}\right)}^{2}$ is not ∩-convex but ∪-convex and $g\left(0\right)=\frac{1}{W^{2}}\neq 0$. This probability functional was used in [31] to classify sleep stages.

Other generalized entropies that are considered below have the form

(14) $$F_{G,g}\left(p_{1},\ldots ,p_{W}\right)=G\left(\sum_{i=1}^{W}g\left(p_{i}\right)\right),$$

where *G* is a continuous *monotonic* function, and *g* is continuous with g(0)=0. By definition, $F_{G,g}$ is also symmetric, and Proposition (iii) holds with the obvious changes. However, the concavity of *g* is not a sufficient condition any more for $F_{G,g}$ to be a generalized entropy. Such is the case of the Rényi entropy $R_{q}$ (Equation (3)); here

(15) $$G\left(u\right)=\frac{1}{1-q}\ln u\;\;\mathrm{and}\;\;g\left(x\right)=x^{q},$$

but g(x) (and, hence, $\sum_{i=1}^{W}g\left(p_{i}\right)$) is not ∩-convex for q>1. Furthermore, note that axiom SK3 requires q>0 for $R_{q}$ to be a generalized entropy.

Since Equation (10) is a special case of Equation (14) (set *G* to be the identity map id(u)=u), we can refer to both cases just by using the notation $F_{G,g}$, as we do hereafter.

We say that two probability distributions $\left\{p_{i}\right\}$ and $\left\{p_{i}^{\prime }\right\}$, 1≤i≤W, are close if

$$∥\left\{p_{i}\right\}-\left\{p_{i}^{\prime }\right\}∥=\sum_{i=1}^{W}\left|p_{i}-p_{i}^{\prime }\right|\leq \delta ,$$

where 0<δ≪1; other norms, such as the two-norm and the max-norm, will do as well since they are all equivalent in the metric sense. A function $F:P\to R^{+}$ is said to be *Lesche-stable* if for all *W* and ϵ>0 there exists δ>0 such that

(16) $$∥\left\{p_{i}\right\}-\left\{p_{i}^{\prime }\right\}∥\leq \delta \;\Rightarrow \;\left|\frac{F\left(\left\{p_{i}\right\}\right)-F\left(\left\{p_{i}^{\prime }\right\}\right)}{F_{\max}}\right|<\epsilon ,$$

where $F_{\max}$$=\max_{\{p_{i}\}\in P}F\left(\left\{p_{i}\right\}\right)$. It follows that

$$\lim_{\delta \to 0}\lim_{W\to \infty }\left|\frac{F\left(\left\{p_{i}\right\}\right)-F\left(\left\{p_{i}^{\prime }\right\}\right)}{F_{\max}}\right|=0.$$

Lesche stability is called *experimental robustness* in [15] because it guarantees that similar experiments performed on similar physical systems provide similar results for the function *F*. According to [16], all admissible systems are Lesche stable.

## 3. Examples of Generalized Entropies

As way of illustration, we put the focus in this section on two classical generalized entropies as well as on some newer ones. The classical examples are the Tsallis entropy and the Rényi entropy because they have extensively been studied in the literature from an axiomatic point of view too. As it turns out, they are unique under some natural assumptions, such as additivity, α-additivity or composability (see below for details). The newer entropies are related to potential applications of the concept of entropy to graph theory [18]. Other examples of generalized entropies are listed in Appendix A for further references.

### 3.1. Tsallis Entropy

A simple way to introduce Tsallis’ entropy as a generalization of the BGS entropy is the following [15]. Given q∈R, define the *q*-logarithm of a real number x>0 as

$$\ln_{q}x=\left\{\begin{array}{cc} \ln x & \mathrm{if}\;q=1,\\ \frac{x^{1-q}-1}{1-q} & \mathrm{otherwise}. \end{array}\right.$$

Note that $\ln_{1}x$ is defined by continuity since $\lim_{q\to 1}\ln_{q}x=\ln x$. If the logarithm in the definition of $S_{BGS}$, Equation (1), is replaced by $\ln_{q}$, then we obtain the Tsallis entropy:

(17) $$T_{q}\left(p_{1},\ldots ,p_{W}\right)=\sum_{i=1}^{W}p_{i}\ln_{q}\left(1/p_{i}\right)=\frac{1}{1-q}\left(\sum_{i=1}^{W}p_{i}^{q}-1\right).$$

As noted before, q>0 for $T_{q}$ to be an admissible generalized entropy.

Alternatively, the definition

$$S_{BGS}\left(p_{1},\ldots ,p_{W}\right)=-\frac{d}{dx}{\left.\sum_{i=1}^{W}p_{i}^{x}\right|}_{x=1}$$

can also be generalized to provide the Tsallis entropy via the *q*-derivative,

$$T_{q}\left(p_{1},\ldots ,p_{W}\right)=-D_{q}{\left.\sum_{i=1}^{W}p_{i}^{x}\right|}_{x=1},$$

where

$$D_{q}f\left(x\right):=\frac{f(qx)-f(x)}{qx-x}.$$

Set qx=x+h, i.e., h=(q−1)x, and let h→0 to check that $D_{1}f\left(x\right)\equiv \lim_{q\to 1}D_{q}f\left(x\right)=df\left(x\right)/dx$.

Although Tsallis proposed his entropy (Equation (17)) in 1988 to go beyond the standard statistical mechanics [12], basically the same formula had already been proposed in 1967 by Havrda and Charvát (with a different multiplying factor) in the realm of cybernetics and control theory [32].

**Figure 1:**
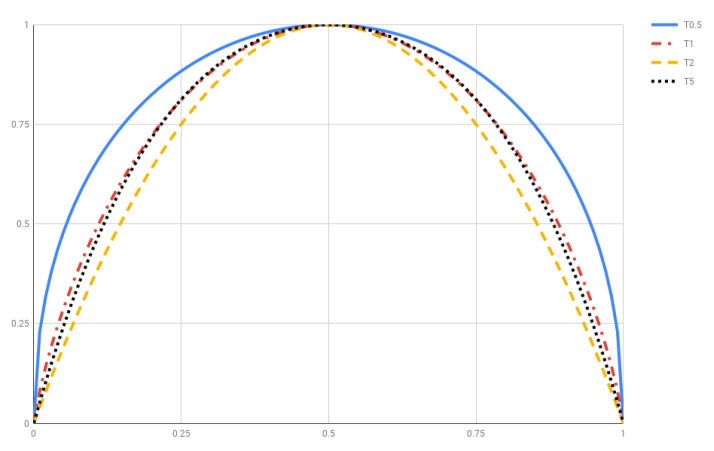
Tsallis entropy $T_{q}\left(p,1-p\right)$ for q=0.5,1,2 and 5.

Some basic properties of $T_{q}$ follow.

(**T1**) $T_{1}=S_{BGS}$ because $\ln_{1}p_{i}=\ln p_{i}$ (or $D_{1}f\left(x\right)=df\left(x\right)/dx$).
(**T2**) $T_{q}$ is (strictly) ∩-convex for q>0. Figure 1 plots $T_{q}\left(p,1-p\right)$ for q=0.5, 1, 2 and 5. Let us mention in passing that $T_{q}$ is ∪-convex for q<0.
(**T3**) $T_{q}$ is Lesche-stable for all q>0 [33,34]. Actually, we stated at the end of Section 2 that all admissible systems are Lesche stable.
(**T4**) $T_{q}$ is not additive but *q*-additive (see Equation (6) or (7) with α replaced by *q*). This property follows from [15]
  $$\ln_{q}xy=\ln_{q}x+\ln_{q}y+\left(1-q\right)\left(\ln_{q}x\right)\left(\ln_{q}y\right).$$
(**T5**) Similar to what happens with the BGS entropy, Tsallis entropy can be uniquely determined (except for a multiplicative positive constant) by a small number of axioms. Thus, Abe [35] characterized the Tsallis entropy by: (i) continuity; (ii) the increasing monotonicity of $T_{q}\left(1/W,\ldots ,1/W\right)$ with respect to *W*; (iii) expansivity; and (iv) a property involving conditional entropies. Dos Santos [36], on the other hand, used the previous Axioms (i) and (ii), *q*-additivity, and a generalization of the grouping axiom (Equation (9)). Suyari [37] derived $T_{q}$ from the first three Shannon–Khinchin axioms and a generalization of the fourth one. The perhaps most economical characterization of $T_{q}$ was given by Furuichi [38]; it consists of continuity, symmetry under the permutation of $p_{1},\ldots ,p_{W}$, and a property called *q*-recursivity. As mentioned in Section 2, Tsallis entropy was recently shown [27] to be the only composable generalized entropy of the form in Equation (10) under some technical assumptions. Further axiomatic characterizations of the Tsallis entropy can be found in [39].

An observable of a thermodynamical (i.e., many-particle) system, say its energy or entropy, is said to be extensive if (among other characterizations), for a large number *N* of particles, that observable is (asymptotically) proportional to *N*. For example, for a system whose particles are weakly interacting (think of a dilute gas), the additive $S_{BGS}$ is extensive, whereas the non-additive $T_{q}$ (q≠1) is non-extensive. The same happens with ergodic systems [40]. However, according to [15], for a non-ergodic system with strong correlations, $S_{BGS}$ can be non-extensive while $T_{q}$ can be extensive for a particular value of *q*; such is the case of a microcanonical spin system on a network with growing constant connectancy [40]. This is why $T_{q}$ represents a physically relevant generalization of the traditional $S_{BGS}$. Axioms SK1–SK3 are expected to hold true also in strongly interacting systems.

Further applications of the Tsallis entropy include astrophysics [41], fractal random walks [42], anomalous diffusion [43,44], time series analysis [45], classification [46,47], and artificial neural networks [48].

### 3.2. Rényi Entropy

A simple way to introduce Rényi’s entropy as a generalization of $S_{BGS}$ is the following [17]. By definition, the BGS entropy of the probability distribution $\left\{p_{1},\ldots ,p_{W}\right\}$ (or of a random variable *X* with that probability distribution) is the *linear* average of the information function

$$I\left(p_{i}\right)=\ln\frac{1}{p_{i}},\;\;1\leq i\leq W,$$

or, equivalently, the expected value of the random variable $\ln\frac{1}{p(X)}$:

$$S_{BGS}\left(p_{1},\ldots ,p_{W}\right)=E_{p}\left[\ln\frac{1}{p(X)}\right]=\sum_{i=1}^{W}p_{i}I\left(p_{i}\right).$$

In the general theory of expected values, for any invertible function ϕ and realizations $x_{1},\ldots ,x_{W}$ of *X* in the definition domain of ϕ, an expected value can be defined as

$$E_{p,\phi }\left[X\right]=\phi^{-1}\left(\sum_{i=1}^{W}p_{i}\phi \left(x_{i}\right)\right).$$

Applying this definition to $\ln\frac{1}{p(X)}$, we obtain

$$E_{p,\phi }\left[\ln\frac{1}{p(X)}\right]=\phi^{-1}\left(\sum_{i=1}^{W}p_{i}\phi \left(I\left(p_{i}\right)\right)\right).$$

If this generalized average has to be additive for independent events, i.e., it has to satisfy Equation (6) with α=1, then

$$\phi \left(x\right)=c_{1}x\;\;\mathrm{or}\;\;\phi \left(x\right)=c_{2}^{(1-q)x}$$

must hold, where $c_{1}$, $c_{2}$ are positive constants, and q>0,q≠1. The first case leads to $S_{BGS}$, Equation (1), after choosing $c_{1}=e$. The second case leads to the Rényi entropy (actually, a one-parameter family of entropies) $R_{q}$, Equation (3), after choosing $c_{2}=e$ as well.

**Figure 2:**
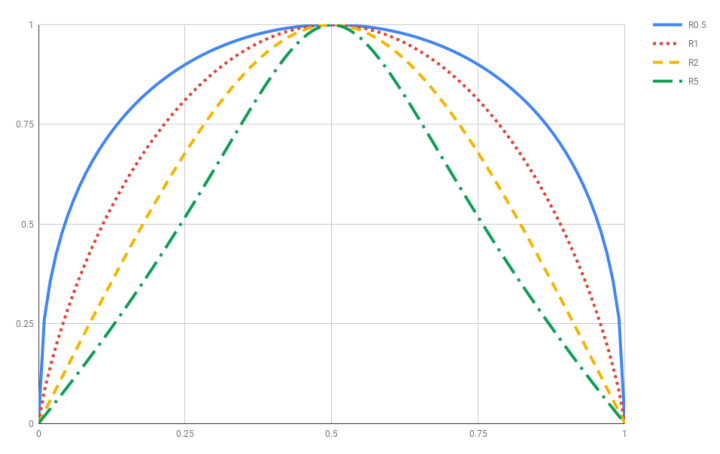
Rényi entropy $R_{q}\left(p,1-p\right)$ for q=0.5,1,2 and 5.

Next, we summarize some important properties of the Rényi entropy.

(**R1**) $R_{q}$ is additive by construction.
(**R2**) $R_{1}\equiv \lim_{q\to 1}R_{q}=S_{BGS}$. Indeed, use L’Hôpital’s Rule to derive
  $$\begin{array}{ccc} \lim_{q\to 1}\frac{1}{1-q}\ln\left(\sum_{i=1}^{W}p_{i}^{q}\right) & = & -\lim_{q\to 1}\frac{d}{dq}\ln\left(\sum_{i=1}^{W}p_{i}^{q}\right)\\ & = & -\lim_{q\to 1}\frac{1}{\sum_{i=1}^{W}p_{i}^{q}}\sum_{i=1}^{W}p_{i}^{q}\ln p_{i}\\ & = & -\sum_{i=1}^{W}p_{i}\ln p_{i}. \end{array}$$
(**R3**) $R_{q}$ is ∩-convex for 0<q≤1 and it is neither ∩-convex nor ∪-convex for q>1. Figure 2 plots $R_{q}\left(p,1-p\right)$ for q=0.5, 1, 2 and 5.
(**R4**) $R_{q}$ is Lesche-unstable for all q>0, q≠1 [49].
(**R5**) The entropies $R_{q}$ are monotonically decreasing with respect to the parameter *q* for any distribution of probabilities, i.e.,
  $$q<q^{\prime }\;\;⟹\;\;R_{q}\geq R_{q^{\prime }}.$$
  This property follows from the formula
  $$-\frac{dR_{q}}{dq}=\frac{1}{{\left(1-q\right)}^{2}}\sum_{i=1}^{W}p_{i}^{\prime }\ln\frac{p_{i}^{\prime }}{p_{i}}=\frac{1}{{\left(1-q\right)}^{2}}D(\left\{p_{i}^{\prime }\right\}∥\{p_{i}\}),$$
  where $p_{i}^{\prime }=p_{i}^{q}/\sum_{k=1}^{W}p_{k}^{q}$, and $D(\left\{p_{i}^{\prime }\right\}∥\{p_{i}\})\geq 0$ is the Kullback–Leibler divergence of the probability distributions $\left\{p_{1}^{\prime },\ldots ,p_{W}^{\prime }\right\}$ and $\left\{p_{1},\ldots ,p_{W}\right\}$. $D(\left\{p_{i}^{\prime }\right\}∥\{p_{i}\})$ vanishes only in the event that both probability distributions coincide, otherwise is positive [26].
(**R6**) A straightforward relation between Rényi’s and Tsallis’ entropies is the following [50]:
  $$T_{q}=\frac{1}{1-q}\left(e^{\left(1-q\right)R_{q}}-1\right)\;\;\mathrm{or}\;\;R_{q}=\frac{1}{1-q}\ln\left(1+\left(1-q\right)T_{q}\right).$$
  However, the axiomatic characterizations of the Rényi entropy are not as simple as those for the Tsallis entropy. See [27,51,52] for some contributions in this regard.

For some values of *q*, $R_{q}$ has particular names. Thus, $R_{0}=\ln W$ is called *Hartley* or *max-entropy*, which coincides numerically with $S_{BGS}$ for an even probability distribution. We saw in (R2) that $R_{q}$ converges to the BGS entropy in the limit q→1. $R_{2}=-\sum_{i=1}^{W}p_{i}^{2}$ is called *collision entropy*. In the limit q→∞, $R_{q}$ converges to the *min-entropy*

$$R_{\infty }\left(p_{1},\ldots ,p_{W}\right)=\min_{1\leq i\leq W}\left(-\ln p_{i}\right)=-\max_{1\leq i\leq W}\ln p_{i}=-\ln\max_{1\leq i\leq W}p_{i}.$$

The name of $R_{\infty }$ is due to property (R5).

Rényi entropy has found interesting applications in random search [53], information theory (especially in source coding [54,55]), cryptography [56], time series analysis [57], and classification [46,58], as well as in statistical signal processing and machine learning [17].

### 3.3. Graph Related Entropies

As part of ongoing work on graph entropy [18], the following generalized entropies are defined:

(18) $$H_{1}\left(p_{1},\ldots ,p_{W}\right)=\sum_{i=1}^{W}\left(1-{\left(p_{i}\right)}^{p_{i}}\right),$$

(19) $$H_{2}\left(p_{1},\ldots ,p_{W}\right)=\prod_{i=1}^{W}\left(2-{\left(p_{i}\right)}^{p_{i}}\right)=\exp\left(\sum_{i=1}^{W}\ln\left(2-{\left(p_{i}\right)}^{p_{i}}\right)\right),$$

and

(20) $$H_{3}\left(p_{1},\ldots ,p_{W}\right)=1+\ln H_{2}\left(p_{1},\ldots ,p_{W}\right)=\sum_{i=1}^{W}\left(p_{i}+\ln\left(2-{\left(p_{i}\right)}^{p_{i}}\right)\right).$$

Note that $H_{1}\left(\ldots ,0,1,0,etc.\right)=0$, while $H_{2}\left(\ldots ,0,1,0,\ldots \right)=H_{3}\left(\ldots ,0,1,0,\ldots \right)=1$. Other oddities of the above entropies include the terms ${\left(p_{i}\right)}^{p_{i}}$ in their definitions, as well as the presence of products instead of sums in the definition of $H_{2}$.

First, $H_{1}$ is of the type in Equation (10) with

(21) $$g_{1}\left(x\right)=\left\{\begin{array}{cc} 0 & \mathrm{if}\;x=0,\\ 1-x^{x} & \mathrm{if}\;0<x\leq 1. \end{array}\right.$$

By definition, g(x) is continuous (even smooth), concave on the interval [0,1], and $g_{1}\left(0\right)=0$. Therefore (see Conditions (C1)–(C3) in Section 2), H1 satisfies the axioms SK1–SK3, hence it is a generalized entropy.

As for $H_{2}$, this probability functional is of the type in Equation (14) with

(22) $$g_{2}\left(x\right)=\left\{\begin{array}{cc} 0 & \mathrm{if}\;x=0,\\ \ln\left(2-x^{x}\right) & \mathrm{if}\;0<x\leq 1, \end{array}\right.$$

and $G\left(u\right)=e^{u}$. To prove that $H_{2}$ is a generalized entropy, note that

$$\ln H_{2}\left(p_{1},\ldots ,p_{W}\right)=\sum_{i=1}^{W}\ln\left(2-{\left(p_{i}\right)}^{p_{i}}\right)$$

satisfies axioms SK1–SK3 for the same reasons as $H_{1}$ does. Therefore, the same happens with $H_{2}$ on account of the exponential function being continuous (SK1), increasingly monotonic (SK2), and univalued (SK3).

Finally, $H_{3}$ is of the type in Equation (10) with

(23) $$g_{3}\left(x\right)=\left\{\begin{array}{cc} 0 & \mathrm{if}\;x=0,\\ x+g_{2}\left(x\right) & \mathrm{if}\;0<x\leq 1. \end{array}\right.$$

Since $H_{3}=1+\ln H_{2}$, it is a generalized entropy because, as shown above, $\ln H_{2}$ satisfies axioms SK1–SK3.

**Figure 3:**
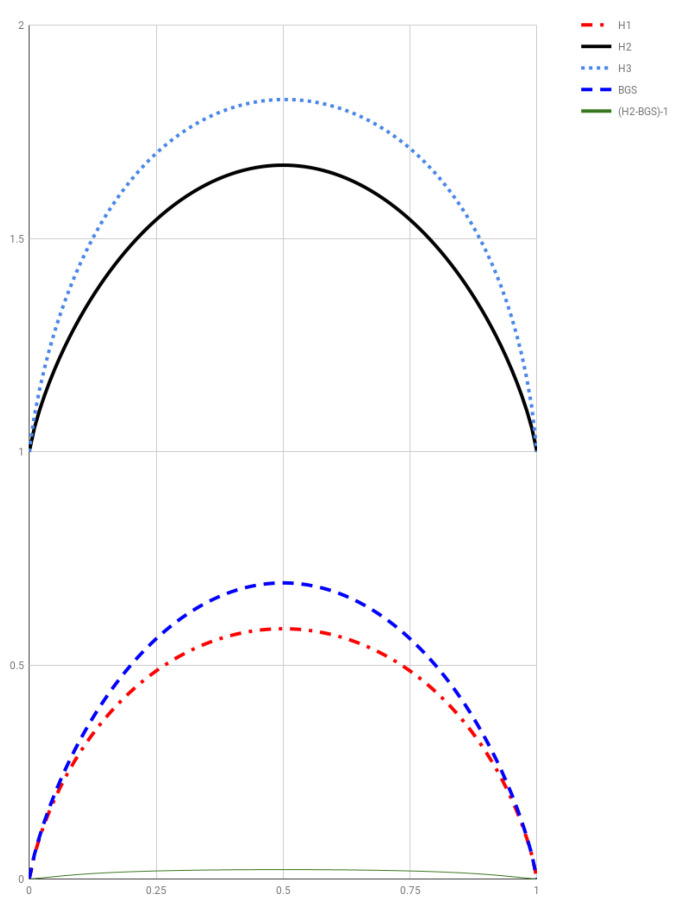
Entropies $H_{i}\left(p,1-p\right)$, i=1,2,3, along with $S_{BGS}\left(p,1-p\right)$ and $H_{2}-S_{BGS}-1$ for comparison.

**Figure 4:**
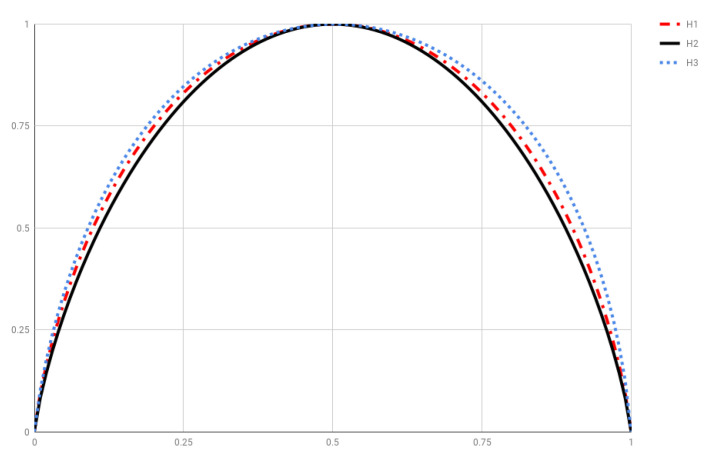
Scaled entropies ${\tilde{H}}_{i}\left(p,1-p\right)$, i=1,2,3, see Equation (24).

Figure 3 depicts $H_{1}\left(p,1-p\right)$, $H_{2}\left(p,1-p\right)$, $H_{3}\left(p,1-p\right)$, along with $S_{BGS}\left(p,1-p\right)$ and $H_{2}-S_{BGS}-1$ for comparison. As a curiosity, let us point out that the scaled versions

(24) $${\tilde{H}}_{i}\left(p,1-p\right)=\frac{H_{i}\left(p,1-p\right)-H_{i}\left(0,1\right)}{H_{i}\left(\frac{1}{2},\frac{1}{2}\right)-H_{i}\left(0,1\right)},$$

(i=1,2,3), see Figure 4, approximate $S_{BGS}\left(p,1-p\right)$ measured in bits very well. In particular, the relative error in the approximation of $S_{BGS}\left(p,1-p\right)$ by ${\tilde{H}}_{2}\left(p,1-p\right)$ is less than $2.9\times 10^{-4}$, so their graphs overlap when plotted.

A further description of the entropies in Equations (18)–(20) is beyond the scope of this section. Let us only mention in this regard that these entropies can be extended into the realm of acyclic directed graphs.

## 4. Hanel–Thurner Exponents

All generalized entropies $F_{G,g}$ group in classes labeled by two exponents (c,d) introduced by Hanel and Thurner [16], which are determined by the limits

(25) $$\lim_{W\to \infty }\frac{F_{G,g}\left(p_{1},\ldots ,p_{\lambda W}\right)}{F_{G,g}\left(p_{1},\ldots ,p_{W}\right)}=\lambda^{1-c}$$

(*W* being as before the cardinality of the probability distribution or the total number of microstates in the system, λ>1) and

(26) $$\lim_{W\to \infty }\frac{F_{G,g}\left(p_{1},\ldots ,p_{W^{1+a}}\right)}{F_{G,g}\left(p_{1},\ldots ,p_{W}\right)}W^{a(c-1)}={\left(1+a\right)}^{d}$$

(a>0). Note that the limit in Equation (26) does not depend actually on *c*. The limits in Equations (25) and (26) can be computed via the asymptotic equipartition property [26]. Thus,

$$F_{G,g}\left(p_{1},\ldots ,p_{\lambda W}\right)\approx G\left(\lambda Wg\left(\frac{1}{\lambda W}\right)\right)$$

and

$$F_{G,g}\left(p_{1},\ldots ,p_{W^{1+a}}\right)\approx G\left(W^{1+a}g\left(\frac{1}{W^{1+a}}\right)\right)$$

asymptotically with ever larger *W* (thermodynamic limit). Set now x=1/W to derive

(27) $$\lim_{x\to 0+}\frac{G\left(\frac{\lambda }{x}g\left(\frac{x}{\lambda }\right)\right)}{G\left(\frac{1}{x}g\left(x\right)\right)}=\lambda^{1-c}$$

and

(28) $$\lim_{x\to 0+}\frac{G\left(\frac{1}{x^{1+a}}g\left(x^{1+a}\right)\right)}{x^{a(c-1)}G\left(\frac{1}{x}g\left(x\right)\right)}={\left(1+a\right)}^{d}.$$

Clearly, the scaling exponents *c*, *d* of a generalized entropy $F_{G,g}$ depend on the behavior of *g* in an infinitesimal neighborhood (0,ε] of 0 (i.e., g(ε) with 0<ε≪1), as well as on the properties of *G* if G≠id. We call (c,d) the *Hanel–Thurner* (HT) *exponents* of the generalized entropy $F_{G,g}$.

When G=id, Equations (27) and (28) abridge to

(29) $$\lim_{x\to 0+}\frac{g(zx)}{g(x)}=z^{c}$$

(after replacing $\lambda^{-1}$ by *z*), and

(30) $$\lim_{x\to 0+}\frac{g(x^{1+a})}{x^{ac}g\left(x\right)}={\left(1+a\right)}^{d},$$

respectively. In this case, 0<c≤1, while *d* can be any real number. If c=1, the concavity of *g* implies d≥0 [16]. The physical properties of admissible systems are uniquely characterized by their HT exponents, i.e., by their asymptotic properties in the limit W→∞ [16]. In this sense, we can also speak of the universality class (c,d).

As way of illustration, we are going to derive the HT exponents of $S_{BGS}$, $T_{q}$ and $R_{q}$.

(**E1**) For the BGS entropy, g(x)=−xlnx (see Equation (11)), so
  $$\frac{g\left(zx\right)}{g\left(x\right)}=\frac{zx\ln(zx)}{x\ln x}=\frac{z\ln z+z\ln x}{\ln x}\to z$$
  as x→0+. Therefore, c=1. Furthermore,
  $$\frac{g\left(x^{1+a}\right)}{x^{ac}g\left(x\right)}=\frac{x^{1+a}\ln x^{1+a}}{x^{a+1}\ln x}=\frac{(1+a)\ln x}{\ln x}=1+a$$
  for all x>0, so d=1.
(**E2**) For the Tsallis entropy, see Equation (12),
  $$g\left(x\right)=\left\{\begin{array}{cc} \frac{1}{1-q}x^{q}+O\left(x\right) & \mathrm{if}\;0<q<1,\\ -\frac{1}{1-q}x+O\left(x\right) & \mathrm{if}\;q>1. \end{array}\right.$$
  It follows readily that (c,d)=(q,0) if 0<q<1, and (c,d)=(1,0) if q>1. Hence, although $\lim_{q\to 1}T_{q}=S_{BGS}$, there is no parallel convergence concerning the HT exponents.
(**E3**) For the Rényi entropy, $g\left(x\right)=x^{q}$ and $G\left(u\right)=\frac{1}{1-q}e^{u}$ (see Equation (15)), so
  $$\frac{G(\frac{\lambda }{x}g\left(\frac{x}{\lambda }\right))}{G(\frac{1}{x}g\left(x\right))}=\frac{\ln\left(\frac{\lambda }{x}{\left(\frac{x}{\lambda }\right)}^{q}\right)}{\ln\left(\frac{1}{x}x^{q}\right)}=\frac{\ln x^{q-1}-\ln\lambda^{q-1}}{\ln x^{q-1}}\to 1$$
  as x→0+ (both for 0≤q≤1 and q≥1). Therefore, c=1. Furthermore,
  $$\frac{G\left(\frac{1}{x^{1+a}}g\left(x^{1+a}\right)\right)}{G\left(\frac{1}{x}g\left(x\right)\right)}=\frac{\ln\left(\frac{1}{x^{1+a}}x^{q(1+a)}\right)}{\ln\left(\frac{1}{x}x^{q}\right)}=\frac{\ln x^{\left(q-1)(1+a\right)}}{\ln x^{q-1}}=1+a$$
  for all x>0, so that d=1. In sum, (c,d)=(1,1) for all *q*.

As for the generalized entropies $H_{1},H_{2}$, and $H_{3}$ considered in Section 3.3, we show in Appendix B that their HT exponents are (1,1), (0,0), and (1,1), respectively. Thus, $H_{1}$ and $H_{3}$ belong to the same universality class as $S_{BGS}$, while the HT exponents of $H_{2}$ and $R_{q}$ (both of the same the type in Equation (14)) are different. Moreover, the interested reader will find in Table 1 of [16] the HT exponents of the generalized entropies listed in Appendix A.

An interesting issue that arises at this point is the inverse question: Given c∈(0,1] and d∈R, is there an admissible system such that its HT exponents are precisely (c,d)? The answer is yes, at least under some restrictions on the values of *c* and *d*. Following [16], we show in Appendix C that, if

(31) $$\begin{array}{cc} d>-1 & \mathrm{for}\;0<c\leq \frac{1}{2},\\ d\geq 1-\frac{1}{c} & \mathrm{for}\;\frac{1}{2}<c\leq 1, \end{array}$$

then the “generalized (c,d)-entropy”

(32) $$S_{c,d}\left(p_{1},\ldots ,p_{W}\right)=eA\sum_{i=1}^{W}\Gamma \left(d+1,1-c\ln p_{i}\right),$$

has HT exponents (c,d). Here, A>0 and Γ is the incomplete Gamma function (Section 6.5 of [59]), that is,

(33) $$\Gamma \left(r,s\right)=\int_{s}^{\infty }t^{r-1}e^{-t}dt\;\;\left(r>0\right).$$

Several application cases where generalized (c,d)-entropies are relevant have been discussed by Hanel and Thurner in [40] (super-diffusion, spin systems, binary processes, and self-organized critical systems) and [60] (aging random walks, i.e., random walks whose transition rates between states are path- and time-dependent).

## 5. Asymptotic Relation between the HT Exponent c and the Diffusion Scaling Exponent

In contrast to “non-interacting” systems, where both the additivity and extensivity of the BGS entropy $S_{BGS}$ hold, in the case of general interacting statistical systems these properties can no longer be simultaneously satisfied, requiring a more general concept of entropy [16,40]. Following [16] (Section 4), a possible generalization of $S_{BGS}$ for admissible systems is defined via the two asymptotic scaling relations in Equations (29) and (30), i.e., the HT exponents *c* and *d*, respectively. These asymptotic exponents can be interpreted as a measure of deviation from the “non-interacting” case regarding the stationary behavior.

### 5.1. The Non-Stationary Regime

In this section, we describe a relation between the exponent *c* and a similar macroscopic measure that characterizes the system in the non-stationary regime, thus providing a meaningful interpretation of the exponent. The non-stationary behavior of a system can possibly be described by the Fokker–Planck (FP) equation governing the time evolution of a probability density function p=p(x,t). In this continuous limit, the generalized entropy $F_{g}$ is assumed to be written as $F_{g}\left[p\left(s\right)\right]=\int g\left(p\left(s\right)\right)ds$, where *g* is asymptotically characterized by Equation (29) and s=s(x) is a time-independent scalar function of the space coordinate *x* (for example, a potential) [61,62].

Going beyond the scope of the simplest FP equation, we consider systems for which the correlation among their (sub-)units can be taken into account by replacing the diffusive term $\partial_{x}^{2}p$ with an effective term $\partial_{x}^{2}\Phi \left[p\right]$, where Φ[p] is a pre-defined functional of the probability density. Φ[p] can be either derived directly from the microscopical transition rules or it may be defined based on macroscopic assumptions. The resulting FP equation can be written as

(34) $$\partial_{t}p\left(x,t\right)=D\beta \partial_{x}\left(p\left(x,t\right)\partial_{x}u\left(x\right)\right)+D\partial_{x}^{2}\Phi \left[p\left(x,t\right)\right],$$

where D,β are constants and u(x) is a time-independent external potential.

For simplicity, hereafter we exclusively focus on one dimensional FP equations. In the special case of Φ[p]=p and no external forces, Equation (34) reduces to the well-known linear diffusion equation

(35) $$\partial_{t}p\left(x,t\right)=D\partial_{x}^{2}p\left(x,t\right).$$

The above equation is invariant under the space-time scaling transformation

(36) $$p\left(x,t\right)=\tau^{-\gamma }p\left(\frac{x}{\tau^{\gamma }},\frac{t}{\tau }\right)$$

with $\gamma =\frac{1}{2}$ [63,64]. This scaling property opens up the possibility of a phenomenological and macroscopic characterization of anomalous diffusion processes [15,44] as well, which correspond to more complicated non-stationary processes described by FP equations in the form of Equation (34) with a non-trivial value of γ. With the help of the transformation in Equation (36), we can also classify correlated statistical systems according to the rate of the spread of their probability density functions over time in the asymptotic limit and, thus, quantitatively describe their behavior in the non-stationary regime.

### 5.2. Relation between the Stationary and Non-Stationary Regime

To reasonably and consistently relate the generalized entropies to the formalism of FP equations—corresponding to the stationary and non-stationary regime, respectively—the functional Φ[p] has to be chosen such that the stationary solution of the general FP equation becomes equivalent to the Maximum Entropy (MaxEnt) probability distribution calculated with the generalized entropies. These MaxEnt distributions can be obtained analogously to the results by Hanel and Thurner in [16,40], where they used standard constrained optimization to find the most general form of MaxEnt distributions, which turned out to be $p\left(\epsilon \right)=E_{c,d,r}\left(-\epsilon \right)$ with

(37) $$E_{c,d,r}\left(x\right)\propto \exp\left[-\frac{d}{1-c}W_{k}\left(B{\left(1-\frac{x}{r}\right)}^{1/d}\right)\right]\;.$$

Here, B,r are constants depending only on the c,d parameters and $W_{k}$ is the *k*th branch of the Lambert-*W* function (specifically, branch k=0 for d≥0 and branch k=1 for d<0). The consistency criterion imposed above accords with the fact that many physical systems tend to converge towards maximum entropy configuration over time, however, it specifies the limits of our assumptions.

Consider systems described by Equation (34) in the absence of external force, i.e.,

(38) $$\partial_{t}p\left(x,t\right)=D\partial_{x}^{2}\Phi \left[p(x,t)\right].$$

By assuming that the corresponding stationary solutions can be identified with the MaxEnt distributions in Equation (37), it can be shown that the functional form of the effective density Φ[p] must be expressed as

(39) $$\Phi \left[p\right]\propto \int_{0}^{p}q\;\partial_{q}^{2}g\left(q\right)dq,$$

where we neglected additive and multiplicative constant factors for the sake of simplicity. Similar implicit equations have already been investigated in [61,62,65]. Once the asymptotic phase space volume scaling relation in Equation (29) holds, it can also be shown that the generalized FP in Equation (38) (with Φ as in Equation (39)) obeys the diffusion scaling property in Equation (36) with a non-trivial value of γ in the p→0 asymptotic limit [66] (assuming additionally the existence of the solution of Equation (38), at least from an appropriate initial condition). A simple algebraic relation between the diffusion scaling exponent γ and the phase space volume scaling exponent *c* can be established [66], which can be written as

(40) $$\gamma =\frac{1}{1+c}.$$

Therefore, this relation between *c* and γ defines families of FP equations which show asymptotic invariance under the scaling relation in Equation (36).

## 6. Conclusions

This review concentrates on the concept of generalized entropy (Section 2), which is relevant in the study of real thermodynamical systems and, more generally, in the theory of complex systems. Possibly the first example of a generalized entropy was introduced by Rényi (Section 3.2), who was interested in the most general information measure which is additive in the sense of Equation (5), with the random variables *X* and *Y* being independent. Another very popular generalized entropy was introduced by Tsallis as a generalization of the Boltzmann–Gibbs entropy (Section 3.1) to describe the properties of physical systems with long range forces and complex dynamics in equilibrium. Some more exotic generalized entropies are considered in Section 3.3, while other examples that have been published in the last two decades are gathered in Appendix A. Our approach was to a great extent formal, with special emphasis in Section 2 and Section 3 on axiomatic formulations and mathematical properties. For expository reasons, applications are mentioned and the original references given as our description of the main generalized entropies progressed, rather than addressing them jointly in a separate section.

An alternative approach to generalized entropies other than the axiomatic one (Section 2) consists in characterizing their asymptotic behavior in the thermodynamic limit W→∞. Hanel and Thurner showed that two scaling exponents (c,d) suffice for admissible generalized entropies, i.e., those entropies of the form in Equation (10) with *g* continuous, concave and g(0)=0 (Section 4); it holds c∈(0,1] and d∈R. As a result, the admissible systems fall in equivalence classes labeled by the exponents (c,d) of the corresponding entropies. Conversely, to each (c,d), there is a generalized entropy with those Hanel–Thurner exponents (see Equation (32)), at least for the most interesting value ranges.

**Figure 5:**
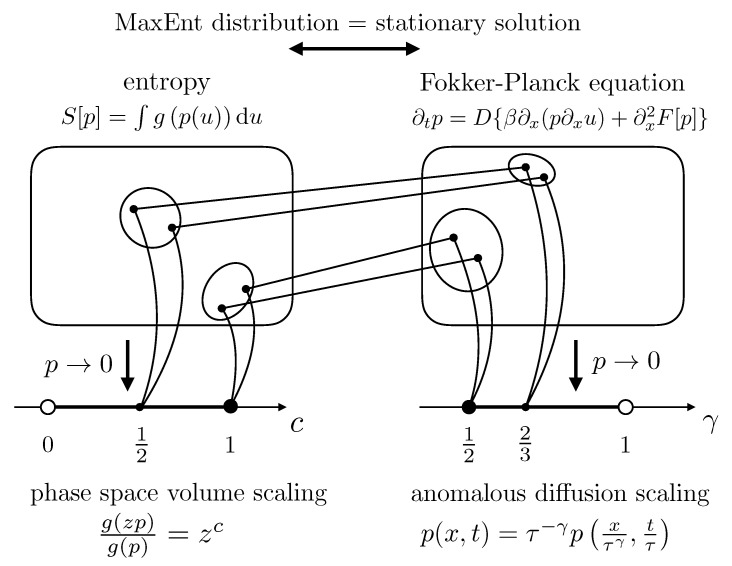
Visual summary of the main result presented in Section 5 schematically depicting the relation between the exponents γ and *c*. Source: [66].

It is also remarkable that, at asymptotically large times and volumes, there is a 1-to-1 relation between the equivalence class of generalized entropies with a given $c\in \left(0,1\right]$ and the equivalence class of Fokker–Planck equations in which the invariance in Equation (36) holds with $\gamma =\frac{1}{1+c}\in \left[\frac{1}{2},1\right)$ (Section 5). This means that the equivalence classes of admissible systems can generally be mapped into anomalous diffusion processes and vice versa, thus conveying the same information about the system in the asymptotic limit (i.e., when p(x,t)→0) [66]. A schematic visualization of this relation is provided in Figure 5. Moreover, the above result can actually be understood as a possible generalization of the Tsallis–Bukman relation [44].

## Appendix A

We list in this appendix further generalized entropies of the form in Equation (10) and the original references (notation as in Table 1 of [16]). Γ(·,·) is the incomplete Gamma function, in Equation (33).

1. $S_{\eta }\left(\left\{p_{i}\right\}\right)=\sum_{i}\Gamma \left(\frac{\eta +1}{\eta },\ln p_{i}\right)-p_{i}\Gamma \left(\frac{\eta +1}{\eta }\right)\;\left(\eta >0\right)$ [67].
2. $S_{\kappa }\left(\left\{p_{i}\right\}\right)=\sum_{i}\frac{p_{i}^{1-\kappa }-p_{i}^{1+\kappa }}{2\kappa }\;\left(0<\kappa <1\right)$ [68].
3. $S_{b}\left(\left\{p_{i}\right\}\right)=\sum_{i}\left(1-e^{bp_{i}}\right)+e^{-b}-1\;\left(b>0\right)$ [69].
4. $S_{E}\left(\left\{p_{i}\right\}\right)=\sum_{i}p_{i}\left(1-e^{\left(p_{i}-1\right)/p_{i}}\right)$ [70].
5. $S_{\beta }\left(\left\{p_{i}\right\}\right)=\sum_{i}p_{i}^{\beta }\ln\left(1/p_{i}\right)\;\left(0<\beta \leq 1\right)$ [71].
6. $S_{\gamma }\left(\left\{p_{i}\right\}\right)=\sum_{i}p_{i}\ln^{1/\gamma }\left(1/p_{i}\right)$ ([15], page 60).

## Appendix B

From Equations (18)–(20), it follows that the functions *g* of $H_{1}$, $H_{2}$, and $H_{3}$ are the following:

$$\begin{array}{ccc} g_{1}\left(\varepsilon \right) & \equiv & 1-\varepsilon^{\varepsilon }\approx -\varepsilon \ln\varepsilon ,\\ g_{2}\left(\varepsilon \right) & \equiv & \ln\left(2-\varepsilon^{\varepsilon }\right)\approx 1-\varepsilon^{\varepsilon }\approx -\varepsilon \ln\varepsilon , \end{array}$$

$$g_{3}\left(\varepsilon \right)\equiv \varepsilon +\ln\left(2-\varepsilon^{\varepsilon }\right)\approx \varepsilon -\varepsilon \ln\varepsilon \approx -\varepsilon \ln\varepsilon .$$

Since $H_{1}$ and $H_{3}$ are generalized entropies of the type in Equation (10), we conclude that both belong to the same class as $S_{BGS}$ (see Equation (11)), hence (c,d)=(1,1).

$H_{2}$ is a generalized entropy of the type in Equation (14) with $G\left(u\right)=e^{u}$. Therefore,

$$\frac{G(\frac{\lambda }{\varepsilon }g_{2}\left(\frac{\varepsilon }{\lambda }\right))}{G(\frac{1}{\varepsilon }g_{2}\left(\varepsilon \right))}\approx \frac{\exp\left(-\frac{\lambda }{\varepsilon }\frac{\varepsilon }{\lambda }\ln\frac{\varepsilon }{\lambda }\right)}{\exp\left(-\ln\varepsilon \right)}=\frac{\lambda /\varepsilon }{1/\varepsilon }=\lambda .$$

Comparison with Equation (27) shows that c=0.

Moreover,

$$\frac{G\left(\frac{1}{\varepsilon^{1+a}}g_{2}\left(\varepsilon^{1+a}\right)\right)}{\varepsilon^{a(c-1)}G\left(\frac{1}{\varepsilon }g_{2}\left(\varepsilon \right)\right)}\approx \frac{\exp\left(-(1+a)\ln\varepsilon \right)}{\varepsilon^{-a}\exp\left(-\ln\varepsilon \right)}=\frac{\varepsilon^{-(1+a)}}{\varepsilon^{-(1+a)}}=1.$$

Comparison with Equation (28) shows that d=0.

## Appendix C

**1.** First, note from Equation (32) that

(A1) $$g_{c,d}\left(x\right)=eA\Gamma \left(d+1,1-c\ln x\right),$$

where the incomplete Gamma function Γ(d+1,1−clnx) exists for d>−1 and all x∈(0,1] (see Equation (33)), with $g_{c,d}\left(0\right)=\lim_{x\to 0+}eA\Gamma \left(d+1,1-c\ln x\right)=0$.

Among Conditions (C1)–(C3) on $g_{c,d}$ (Section 2), for the entropy $S_{c,d}$ in Equation (32) to be admissible, only concavity (Condition (C2)) needs to be checked. Since

$$\begin{array}{ccc} \frac{d^{2}}{dx^{2}}g_{c,d}\left(x\right) & = & eA\frac{d^{2}}{dx^{2}}\Gamma \left(d+1,1-c\ln x\right)\\ & = & eA{\left(\frac{c}{x}\right)}^{2}e^{-1+c\ln x}{\left(1-c\ln x\right)}^{d-1}\times \\ & & \times \left(1-\frac{1}{c}+\left(1-c\right)\ln x-d\right), \end{array}$$

it holds $g_{c,d}^{\prime \prime }\left(x\right)\leq 0$ if and only if $d\geq 1-\frac{1}{c}+\left(1-c\right)\ln x,$ where −∞<(1−c)lnx≤0 for each c∈(0,1] and x∈[0,1]. Therefore, $g_{c,d}^{\prime \prime }\left(x\right)\leq 0$ for all x∈[0,1] if and only if $d\geq 1-\frac{1}{c}$, where $-\infty <1-\frac{1}{c}\leq 0$. On the other hand, d>−1 for the integral Γ(d+1,1−clnx) to exist. Both restrictions together lead then to the condition in Equation (31) on *d* for $S_{c,d}$ to be a generalized entropy.

**2.** Use the asymptotic approximation 6.5.32 of [59]

(A2) $$\begin{array}{ccc} \Gamma (d+1,1-c\ln\varepsilon ) & = & {\left(1-c\ln\varepsilon \right)}^{d}e^{c\ln\varepsilon -1}+O\left(\frac{1}{\ln\varepsilon }\right)\\ & \approx & e^{-1}\varepsilon^{c}{\left(1-c\ln\varepsilon \right)}^{d} \end{array}$$

(d>−1, 0<c≤1) to obtain the leading approximation of $g_{c,d}\left(x\right)$ in an infinitesimal neighborhood of 0:

(A3) $$g_{c,d}\left(\varepsilon \right)\approx A\varepsilon^{c}{\left(1-c\ln\varepsilon \right)}^{d}\approx Ac^{d}\varepsilon^{c}{\left(\ln\frac{1}{\varepsilon }\right)}^{d}.$$

Using Equation (A3), the following can be derived:

(A4) $$\frac{g_{c,d}\left(z\varepsilon \right)}{g_{c,d}\left(\varepsilon \right)}\approx \frac{z^{c}\varepsilon^{c}{\left(\ln\frac{1}{z\varepsilon }\right)}^{d}}{\varepsilon^{c}{\left(\ln\frac{1}{\varepsilon }\right)}^{d}}=z^{c}\frac{{\left(\ln\frac{1}{z}+\ln\frac{1}{\varepsilon }\right)}^{d}}{{\left(\ln\frac{1}{\varepsilon }\right)}^{d}}\approx z^{c}$$

(see Equation (29)) and

(A5) $$\frac{g_{c,d}\left(\varepsilon^{1+a}\right)}{\varepsilon^{ac}g_{c,d}\left(\varepsilon \right)}=\frac{\varepsilon^{c(1+a)}{\left(1+a\right)}^{d}{\left(\ln\frac{1}{\varepsilon }\right)}^{d}}{\varepsilon^{ac}\varepsilon^{c}{\left(\ln\frac{1}{\varepsilon }\right)}^{d}}={\left(1+a\right)}^{d}$$

(see Equation (30)).

**3.** From Equation (A3), we obtain

(A6) $$g_{1,1}\left(\varepsilon \right)\approx -A\varepsilon \ln\varepsilon$$

(see Example (E1)) and,

(A7) $$g_{c,0}\left(\varepsilon \right)\approx A\varepsilon^{c}$$

(see Example (E2)). Set $A={\left(1-c+cd\right)}^{-1}$ [16] in Equations (A6) and (A7) to reproduce the *g* functions of $S_{BGS}$ (A=1) and $T_{c}$, 0<c<1 ($A=\frac{1}{1-c}$), respectively.
